# Clean Indoor Air Regulation and Incidence of Hospital Admissions for Acute Coronary Syndrome in Kanawha County, West Virginia

**Published:** 2011-06-15

**Authors:** Rahul Gupta, Robert H. Anderson, Juhua Luo, Anita Ray

**Affiliations:** Kanawha Charleston Health Department, West Virginia University School of Medicine and University of Charleston, Charleston, West Virginia; West Virginia University, Prevention Research Center, Mary Babb Randolph Cancer Center; West Virginia University, Mary Babb Randolph Cancer Center, Morgantown, West Virginia; Kanawha Charleston Health Department, Charleston, West Virginia

## Abstract

**Introduction:**

Secondhand smoke is a risk factor for coronary heart disease. Laws and regulations prohibiting smoking in public areas and workplaces can reduce rates of acute myocardial infarction. Our objective was to describe hospital admission rates for acute coronary events, based on smoking status, diabetes status, and sex, in the presence of a long-standing (2000-2008) county clean indoor air regulation (CIAR). We also examined the effect of making restaurants completely smoke-free.

**Methods:**

We obtained hospital admission data for acute coronary syndrome (ACS) and acute myocardial infarction from all acute care hospitals serving Kanawha County, West Virginia, for 2000 through 2008. A CIAR was enacted in 1995 and revised in 2000 and 2003. We performed descriptive analyses on hospital admission rates of ACS over time and present these data by sex, age group, smoking status, and medical history of diabetes.

**Results:**

The incidence of hospital admissions for ACS consistently declined during the period studied. This change was most pronounced among nonsmokers, people without diabetes, and women, compared with their respective counterparts. Similar benefits occurred for male smokers when the CIAR was revised to make restaurants completely smoke-free in 2004.

**Conclusions:**

In the presence of a CIAR, a consistent decline in incidence of hospital admissions for ACS can be demonstrated. However, the benefits derived may be disproportionately affected by smoking status, diabetes status, and sex.

## Introduction

Secondhand smoke was established as a cause of lung disease in nonsmokers in 1986 (1). Subsequently, other diseases and adverse effects of secondhand smoke were established, including increased risk for coronary heart disease (CHD) (2). Specifically, secondhand smoke exposure increases CHD risk by 25% to 30% (3). These risks are attributed to various mechanisms including but not limited to endothelial dysfunction and arterial stiffness, increased oxidative stress, reduced heart rate variability, and increased insulin resistance (4).

All levels of US government have been slow to provide the public with comprehensive clean indoor air policies (5). The greatest obstacle to making fundamental societal changes is not funding but the lack of political will (6). In 2009, West Virginia (along with Kentucky) had the highest rate of adult smokers in the nation, 26% (7). However, because of the autonomous nature of local boards of health in West Virginia, only 18 of its 55 counties currently have comprehensive indoor air regulations. The failure to provide this protection places people at risk.

In the first published study of the effect of a smoking ban on heart disease rates, following legislation in Helena, Montana, that required smoke-free workplaces and public places, a significant drop in acute myocardial infarction (AMI) was observed; this reduction ended after 6 months when the ban was repealed (8). Similarly, AMI reductions were found when smoking prohibitions were implemented in 4 other US jurisdictions (9-12) and in Canada (13), Italy (14-16), and Scotland (17). A meta-analysis of 11 reports from 10 study locations demonstrated a mean AMI decrease of 14% after smoking bans were implemented. The effect was most pronounced for younger adults (18). Researchers projected that 156,400 AMIs would be prevented each year if comprehensive smoking regulations were launched in the United States.

In October 2009, the Institute of Medicine concluded that sufficient evidence exists to infer a causal relationship between secondhand smoke exposure and increased risks of CHD illness and death among both men and women and that a decrease in secondhand smoke exposure decreases the risk of AMI (3). However, insufficient evidence exists to determine whether the beneficial effects vary by smoking status, diabetes status, and sex. Whether the decline in hospital admissions for such acute cardiac events is maintained over longer periods is unknown. Therefore, the objective of our study was to describe hospital admission rates over time (2000-2008) for acute coronary events, by smoking status, diabetes status, and sex, in the presence of an existing county-wide clean indoor air regulation (CIAR or regulation). We also examined the effect of making restaurants completely smoke-free.

## Methods

We retrospectively studied electronic hospital records to investigate the effect of a county regulation on public health. Since all data were de-identified, West Virginia University's institutional review board exempted the study from review.

### Setting

Kanawha County is the most populated county in West Virginia and is home to the state capital, Charleston. The 2009 US Census estimated its population at 191,663 (approximately 89% white and 8% African American) (19).

Effective May 22, 1995, a modest smoking regulation was enacted by the Kanawha-Charleston Board of Health, prohibiting smoking in all enclosed public places in Kanawha County. At the time, restaurants were allowed to designate up to 50% of their seating capacity as smoking areas. On July 20, 2000, the CIAR was modified to increase penalties for violations. On April 3, 2003, a revised regulation prohibited smoking in all restaurants and at most worksites. However, to come into compliance, the regulation allowed several businesses an exemption until January 1, 2004.

### Data collection

We obtained data from the 3 major acute care hospitals that serve Kanawha County, including the largest in the state. We examined the following diagnostic codes from the *International Classification of Diseases, Ninth Revision, Clinical Modification* (ICD-9-CM) (www.cdc.gov/nchs/icd9.htm): 410 (410.0-410.9), 411.1, 411.81, 411.89, 413.0, 413.1, and 413.9. Data inclusion criteria were all patients (de-identified) admitted from January 1, 2000, through September 30, 2008, with a primary ICD-9-CM diagnostic code of myocardial infarction, non-ST segment elevation myocardial infarction, or unstable angina (together termed ACS); Kanawha County residents; and patients who were aged 18 years or older on the date of hospital admission.

For each Kanawha County resident, basic information for each patient included the date of hospital admission/discharge, diagnostic code, age, sex, race, associated medical history such as diabetes, and smoking status (whether they smoked in the past year).

### Statistical analysis

We performed descriptive analyses on the number of hospital admissions for ACS over time by sex, age group, smoking status, and patients' medical history of diabetes. We divided the number of hospital admissions by Kanawha County's age- and sex-specific population to calculate ACS hospital admission rates. We estimated age-adjusted rates by using year 2000 US census data as standard population.

We defined the comprehensive CIAR as starting on January 1, 2004. We fitted a Poisson regression model on the data to assess the effect of the CIAR (coded 0 before 2004 and 1 afterward) on the hospital admission of ACS, adjusting for age (18-49 y, 50-59 y, 60-69 y, 70-79 y, and ≥80 y), and we treated the ordered code 1-5 as a continuous variable; sex (female, male), year as a continuous variable, season (spring: March-May; summer: June-August; fall: September-November; and winter: December-February, with spring as reference); tobacco (no, yes), and history of diabetes (no, yes). Because hospital admission for ACS may have a seasonal effect, we adjusted for seasons in our model. We also assessed the impact of the CIAR stratified by sex and smoking status after adjusting for age, year, season, and history of diabetes.

We added interactions between calendar year and important variables (smoking status, diabetes status, dates of the regulations) into the model to examine whether the slope of the time trend would vary by different populations, including the enactment of the regulation. Finally, we also examined data for AMI (coded as 410.0-410.9) similar to the analysis performed for ACS.

## Results

Overall, we included 14,245 hospital patients who were admitted for ACS between January 1, 2000, and September 30, 2008 (mean age, 66 y). Of them, 8,075 (57%) patients were men; 3,633 (26%) were smokers, and 5,048 (35%) had diabetes. The number of hospital admissions of ACS per year declined over the entire period, from 1,949 patients in 2000 to 1,208 in 2008. This decline was most pronounced among nonsmokers, women, and adults without diabetes.

**Figure 1:**
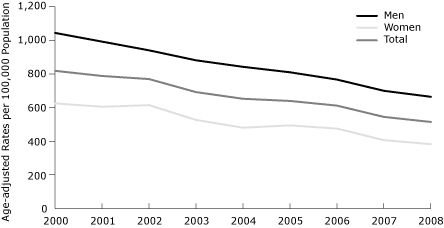
Age-adjusted rates of hospital admissions for acute coronary syndrome, by sex, 2000-2008. Data were obtained from the 3 general hospitals serving Kanawha County, West Virginia, and age-adjusted rates were calculated by using 2000 US Census data as a standard population.

**Figure 2:**
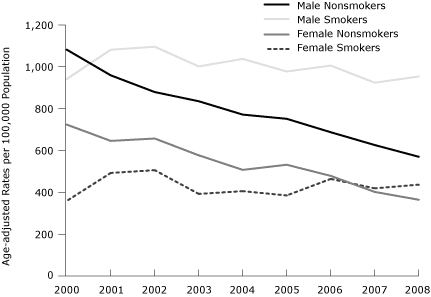
Age-adjusted rates of hospital admissions for acute coronary syndrome, by sex and smoking status, 2000-2008. Data were obtained from the 3 general hospitals serving Kanawha County, West Virginia, and age-adjusted rates were calculated by using 2000 US Census data as a standard population.

**Figure 3:**
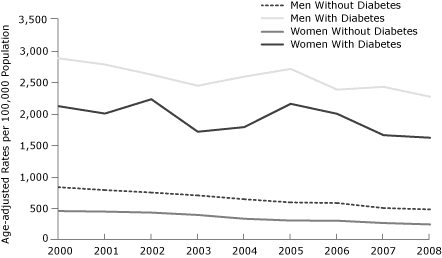
Age-adjusted rates of hospital admissions for acute coronary syndrome, by sex and diabetes status. Data were obtained from the 3 general hospitals serving Kanawha County, West Virginia, and age-adjusted rates were calculated by using 2000 US Census data as a standard population.

The cumulative decrease in the age-adjusted ACS hospitalization rates between 2000 and 2008 was 37% overall, 36% for men and 39% for women. The patterns of decline over time were similar for men and women (Figure 1). However, when we stratified the age-adjusted rates of hospital admissions for ACS by smoking status, the observed decline of age-adjusted hospital admission rates for ACS was significant only among nonsmokers (both men and women) (Figure 2). Declines in the age-adjusted rates for hospital admission of ACS were also noted by diabetes status (Figure 3).

**Table:** 

**Year**	**Men**	**Women**	**Total**
2000	1,044	625	818
2001	992	604	788
2002	940	614	770
2003	881	526	692
2004	842	480	652
2005	810	494	639
2006	767	475	612
2007	700	406	544
2008	664	382	514

**Table:** 

**Year**	**Nonsmoking Men**	**Smoking Men**	**Nonsmoking Women**	**Smoking Women**
2000	1,082	942	724	359
2001	959	1,082	646	493
2002	879	1,096	658	506
2003	835	1,002	577	393
2004	771	1,038	507	406
2005	752	978	532	385
2006	687	1,006	479	464
2007	626	924	402	419
2008	570	954	365	437

**Table:** 

**Year**	**Men Without Diabetes**	**Men With Diabetes**	**Women Without Diabetes**	**Women With Diabetes**
2000	827	2,871	448	2,112
2001	781	2,773	439	1,995
2002	742	2,612	424	2,222
2003	696	2,436	386	1,706
2004	636	2,580	326	1,780
2005	585	2,703	297	2,149
2006	5761	2,374	295	1,991
2007	496	2,418	258	1,652
2008	474	2,262	236	1,612

A Poisson regression model for the main effect of variables of interest revealed that the incidence of hospital admissions for ACS increased significantly with age and decreased with calendar year (6% decrease per year; 95% confidence interval [CI], 4%-8%). Autumn and winter have significantly lower hospital admission rates for ACS compared with spring. The likelihood of hospital admissions for ACS was significantly lower among nonsmokers, people without diabetes, and women.

We did not find additional significant change between, before, and after the removal of smoking areas in restaurants (the key change in the CIAR revision that took effect January 1, 2004) after accounting for the sustainable decline of ACS hospitalizations since the 2000 regulation revision (Table 1).

When we stratified the data by sex and smoking status, the results for the main model among nonsmokers were similar. Among female smokers, other than age and diabetes, we found no significant effect for any other factors. Among male smokers, there was a significant decline in time trend (7% decrease per year; 95% CI, 0.4%-12%) in hospital admission rates for ACS after the revised version of the 2003 CIAR (effective January 1, 2004), but no change over time before the revision (Table 2). In addition, we performed similar analysis for AMI and found that results were similar (data not shown).

## Discussion

To our knowledge, this is the first study to describe a pattern of consistent decline in the rates of hospital admissions for ACS during an 8-year period (000-2008) in a community with an existing CIAR. During this period, a significant drop in ACS hospital admission rates was observed for nonsmokers, people without diabetes, and women. However, this benefit did not reach significance for smokers. Additionally, effective January 1, 2004, when the Kanawha County regulation was further strengthened by making restaurants completely smoke-free (by removal of smoking areas in restaurants), male smokers were able to attain similar gains. Our findings indicate that by eliminating smoking areas at restaurants, bars, airports, and other public places and making them completely smoke-free, we may be able to decrease additionally the number of acute coronary events among male smokers. As expected, our study reveals that people who do not have diabetes benefit more from reduced ACS hospital admission rates regardless of sex and smoking status.

We found no existing data describing the pattern of hospital admission rates for ACS in the presence of an existing CIAR over long periods. To date, only 2 of the studies have presented data on the smoking status of people affected by smoking prohibitions and have conducted analysis in nonsmokers (12,17). Although both studies found a significant decline in the number of admissions among nonsmoking patients after implementation of smoke-free legislation, only 1 also found fewer admissions for smokers (17). However, each study had limitations. Neither of the studies evaluated whether such prohibitions differentially affect men and women. The first study, by Seo and Torabi, had a very small sample size, lasted less than 2 years, included only nonsmoking patients in analysis, excluded many high-risk patients, and provided no information on age (12). The second study, by Pell et al, was also limited by less than 2 years of data analyzed, including only 10 months after implementation of the legislation in Scotland (17).

We recognize that the risk of ACS falls rapidly after smoking cessation (20). In previous research, community-level studies have found that a reduction in secondhand smoke exposure after enactment of ordinances reduces hospital admissions for ACS from a range of 11% to 40% (8,14-17). A random-effects meta-analysis and a meta-regression of several studies to measure AMI reductions in affected communities found a reduction of approximately 15% in hospital ACS admission rates during the first year, and continuing exponential declines reaching approximately 36% in the 3 years following the implementation of strong smoke-free legislation (21). However, there is a paucity of data evaluating the long-term benefits of such legislation on ACS hospital admission rates. Although some recent data of a longer study from Canada demonstrated a consistent decrease in crude rates for hospital admissions due to various cardiovascular and respiratory conditions (22), the study did not delineate individual smoking status. Therefore, although the initial CIAR in Kanawha County was enacted in 1995, we conducted our analysis from 2000 through 2008 to evaluate whether we could identify any cardiovascular benefits in the community in terms of declines in hospital admission rates for ACS. Our results demonstrate a sustained decline in the hospital admission rates from ACS among nonsmokers in Kanawha County, West Virginia. Although this decline may not be conclusively linked directly to the CIAR, a significant benefit in the rate of ACS admissions for male smokers occurred after the regulations were strengthened in 2004 to remove all smoking areas from restaurants.

Our study should be interpreted in light of several limitations. As with many of the past studies mentioned herein, our analysis is a retrospective study of hospital records; therefore, we did not have a closed study population. Our study population was theoretically free to travel from Kanawha County to other neighboring counties where a comprehensive CIAR was not in place. Despite some hospitals in our county being tertiary care centers and therefore serving residents of nearby counties, we restricted our data collection to residents of Kanawha County. Another major limitation of the study is the absence of a control population. Although several studies have used the same population but measured the effect before and after implementation of the ordinance, this was not possible in our case because our starting point of study was 5 years after the implementation of the CIAR. An external control population would have been ideal. However, no comparable population was available. Smoking status of participants was captured at the time of hospital admission on a voluntary, self-report basis. Although unvalidated statements of smoking status are problematic, the likelihood of being untruthful in disclosing smoking status when being admitted for ACS is very low (23).

Other interventions may have affected the outcomes in our population and may serve as potential limitations of the study. First, we evaluated whether there were significant changes at local or state level in prevalence of diseases that may be considered risk factors for coronary artery disease (CAD) during this period. Specifically, we studied the Centers for Disease Control and Prevention's Behavioral Risk Factor Surveillance System and Selected Metropolitan/Micropolitan Area Risk Trends (SMART) data to evaluate the prevalence trends for obesity, diabetes, hypercholesterolemia, hypertension, and physical activity (24). For each of the factors, the rates either increased or remained stable during the period of study. Additionally, a net increase in the prevalence of CAD rates was noted in the state of West Virginia. Second, smoking prevalence remained unchanged in the state from 2000 to 2008 (26% vs 27%). For Kanawha County, although smoking rates decreased from 2002 to 2008 (32% vs 24%), taking CIs into account, the change was not significant (24). Hence, any reduction in ACS admissions cannot be accounted for by a reduction in smoking. Third, our data did not differentiate between nonsmokers and former smokers who remained abstinent more than 1 year. We were also not able to account for those former smokers who may have switched to using smokeless tobacco. However, this is likely to be a small number of people. Fourth, in 2003, West Virginia increased its state tax on tobacco from 17 cents to 55 cents per pack. However, state tobacco tax revenue data indicate that the increase had no effect on sales (25), and considering the lack of significant difference in prevalence, we can dismiss the notion that changes in ACS can be attributed to a decline in smoking.

West Virginia has no state law pertaining to clean indoor air, although local boards of health have the authority to enact smoking regulations. All 55 counties in the state have some form of smoking regulation. Only 18 counties (including Kanawha County) have a comprehensive regulation that includes all worksites. In our review, we found Kanawha County's regulation to be most comprehensive and evenly enforced with high rates of compliance. We recommend that future studies measure baseline data on secondhand smoke exposure in the study population to evaluate the cause-effect relationship of lowering exposure to secondhand smoke in both the short term and the long term. Such studies should include both smokers and nonsmokers. We also recommend that such studies be followed by an analysis of potential cost savings resulting from long-term declines in rate of ACS-related hospital admissions.

In conclusion, our results demonstrate that from 2000 through 2008, the rate of hospital admissions for ACS has consistently declined in Kanawha County in the presence of an existing CIAR. However, these beneficial effects have not been evenly distributed across all populations. Men and women who do not smoke and people who do not have diabetes derive the greatest benefits. Additional benefits for male smokers can be derived from further enhancing the regulations by removal of all smoking areas in restaurants.

## Figures and Tables

**Table 1:** Change in Rate of Acute Coronary Syndrome Hospital Admissions by Selected Variables, Kanawha County, West Virginia

**Variable**	Coefficient (95% CI)	Exponential of Coefficients (95% CI)	*P* Value
**Patient characteristic**			
Age	0.35 (0.33 to 0.37)	1.41 (1.39 to 1.45)	<.001
Year of admission	−0.06 (−0.08 to −0.04)	0.94 (0.92 to 0.96)	<.001
Female	−0.45 (−0.50 to 0.41)	0.64 (0.60 to 0.66)	<.001
**Season**			
Autumn	−0.10 (−0.16 to −0.03)	0.90 (0.85 to 0.97)	.003
Summer	−0.04 (−0.11 to 0.02)	0.96 (0.90 to 1.02)	.18
Winter	−0.07 (−0.13 to −0.002)	0.93 (0.88 to 1.00)	.04
**Health status**			
No tobacco use	−0.25 (−0.31 to −0.20)	0.78 (0.73 to 0.82)	<.001
No diabetes	−1.66 (−1.70 to −1.61)	0.19 (0.18 to 0.20)	<.001
**Regulation change**	0.02 (−0.08 to 0.11)	1.02 (0.92 to 1.12)	.12

Abbreviation: CI, confidence interval.

**Table 2:** Change in Rate of Acute Coronary Syndrome Hospital Admissions by Patient Characteristics, Season, Health Status, and Regulation Status, Based on Smoking Status and Sex, Kanawha County, West Virginia^a^

**Variable**	Nonsmoking Men			Nonsmoking Women		

	Coefficients (95% CI)	Exponential of coefficients (95% CI)	*P* Value	Coefficients (95% CI)	Exponential of Coefficients (95% CI)	*P* Value
**Patient characteristic**						
Age	0.42 (0.39 to 0.45)	1.52 (1.48 to 1.57)	<.001	0.42 (0.39 to 0.45)	1.52 (1.48 to 1.57)	<.001
Year	−0.09 (−0.13 to 0.04)	0.91 (0.88 to 0.96)	<.001	−0.07 (−0.12 to −0.02)	0.93 (0.88 to 0.98)	.007
**Season**						
Autumn	−0.13 (−0.23 to 0.03)	0.87 (0.79 to 0.97)	.01	−0.08 (−0.20 to 0.04)	0.92 (0.82 to 1.04)	.17
Summer	−0.07 (−0.17 to 0.03)	0.93 (0.84 to 1.03)	.15	−0.02 (−0.14 to 0.10)	0.98 (0.87 to 1.11)	.76
Winter	−0.11 (−0.21 to 0.005)	0.90 (0.81 to 1.00)	.04	−0.05 (−0.16 to 0.07)	0.95 (0.85 to 1.07)	.45
**No diabetes**	−1.65 (−1.72 to −1.57)	0.19 (0.18 to 0.21)	<.001	−1.80 (−1.89 to -1.71)	0.17 (0.15 to 0.18)	<.001
**Regulation change^b^**	0.04 (−0.20 to 0.27)	1.04 (0.82 to 1.31)	.75	0.05 (−0.23 to 0.32)	1.05 (0.79 to 1.38)	.74
**Regulation-year**	0.01 (−0.05 to 0.07)	1.01 (0.95 to 1.07)	.68	−0.005 (−0.07 to 0.06)	1.00 (0.93 to 1.06)	.89

**Variable**	**Smoking Men**			**Smoking Women**		

	**Coefficients (95% CI)**	**Exponential of Coefficients (95% CI)**	***P* Value**	**Coefficients (95% CI)**	**Exponential of Coefficients (95% CI)**	***P* Value**

**Patient characteristic**						
Age	−0.02 (−0.06 to 0.02)	0.98 (0.94 to 1.02)	.31	0.12 (0.08 to 0.16)	1.13 (1.08 to 1.17)	<.001
Year	0.03 (−0.02 to 0.08)	1.03 (0.98 to 1.08)	.22	0.03 (−0.03 to 0.10)	1.03 (0.97 to 1.11)	.28
**Season**						
Autumn	−0.09 (−0.20 to 0.02)	0.91 (0.82 to 1.02)	.12	−0.03 (−.16 to 0.11)	0.97 (0.85 to 1.12)	.71
Summer	−0.02 (−0.13 to 0.09)	0.98 (0.88 to 1.09)	.68	−0.05 (−0.18 to 0.08)	0.95 (0.84 to 1.08)	.47
Winter	−0.04 (−0.16 to 0.06)	0.96 (0.85 to 1.06)	.39	0.01 (−0.13 to 0.14)	1.01 (0.88 to 1.15)	.89
**No diabetes**	−1.27 (−1.36 to −1.18)	0.28 (0.26 to 0.31)	<.001	−1.68 (−1.79 to −1.58)	0.19 (0.17 to 0.21)	<.001
**Regulation change**	0.12 (−0.13 to 0.37)	1.13 (0.88 to 1.45)	.33	0.02 (−0.29 to 0.33)	1.02 (0.75 to 1.39)	.91
**Regulation-year^b^**	−0.07 (−0.13 to −0.004)	0.93 (0.88 to 1.00)	.04	−0.05 (−0.13 to 0.03)	0.95 (0.88 to 1.03)	.18

Abbreviation: CI, confidence interval.

a In each Poisson model, we defined the variables as age (1: 18-49, 2: 50-59, 3: 60-69, 4: 70-79, and 5: ≥80 y) In each Poisson model, we defined the variables as age (1: 18-49, 2: 50-59, 3: 60-69, 4: 70-79, and 5: ≥80 y), and the ordered code was treated as a continuous variable); year (1-9 for 2000-2008, treated as a continuous variable); season (spring, March-May; summer, June-August; fall, September-November; and winter, December-February, with spring as reference); no diabetes (compared with diabetes); and regulation (0: 2000-2003, 1: 2004-2008).

b The relationship between regulation and year.
